# Estimates of the True Number of Cases of Pandemic (H1N1) 2009, Beijing, China

**DOI:** 10.3201/eid1611.100323

**Published:** 2010-11

**Authors:** Xiaoli Wang, Peng Yang, Holly Seale, Yi Zhang, Ying Deng, Xinghuo Pang, Xiong He, Quanyi Wang

**Affiliations:** Author affiliations: Beijing Center for Disease Prevention and Control, Beijing, People’s Republic of China (X. Wang, P. Yang, Y. Zhang, Y. Deng, X. Pang, X. He, Q. Wang);; Capital Medical University School of Public Health and Family Medicine. Beijing (X. Wang, P. Yang, Y. Zhang, Y. Deng, X. Pang, X. He, Q. Want);; University of New South Wales, Sydney, New South Wales, Australia (H. Seale)

**Keywords:** Monte Carlo approach, multiplier model, influenza, pandemic (H1N1) 2009, estimates, dispatch

## Abstract

During 2009, a total of 10,844 laboratory-confirmed cases of pandemic (H1N1) 2009 were reported in Beijing, People’s Republic of China. However, because most cases were not confirmed through laboratory testing, the true number is unknown. Using a multiplier model, we estimated that ≈1.46–2.30 million pandemic (H1N1) 2009 infections occurred.

Infection with a novel swine-origin influenza A (H1N1) virus, currently named pandemic (H1N1) 2009 virus, first occurred in the United States and Mexico in early April 2009 (*1*,*2*) and then rapidly spread to other regions of the world. As the outbreak expanded, laboratory testing of persons with suspected cases became increasingly impractical, extremely resource intensive, and was discontinued. We assume, therefore, that the number of laboratory-confirmed cases represents only a small fraction of the actual number of infections (*3*–*5*). In this study, we used a multiplier model to estimate the true number of cases of pandemic (H1N1) 2009 in Beijing, People’s Republic of China.

## The Study

To estimate the prevalence of pandemic (H1N1) 2009 in the United States, the US Centers for Disease Control and Prevention (CDC) developed a software program (Impact2009, version 1.0) (*6*) based on the Monte Carlo approach and the multiplier model. Although this simple and useful program can be used to estimate the true number of cases in the United States, it may not be so readily applied to other countries because of uncertainties in the model parameters. To account for these uncertainties, in this study we decided to alter the way in which the baseline data assumptions were calculated. For example in the original CDC model, the prevalence was calculated on the basis of the laboratory-confirmed case data. In contrast, we calculated the baseline case number by multiplying the reported number of influenza-like illness (ILI) cases in secondary and tertiary hospitals by the positive rate of pandemic (H1N1) 2009 among ILI cases. We obtained this information from the Beijing influenza surveillance system, which encompasses data on ILI cases from all secondary and tertiary hospitals (levels 2, 3), and virologic surveillance data (*7*).

From the virologic surveillance data, we determined that positive cases of pandemic (H1N1) 2009 were identified through August 3, 2009. From this finding, we used 2 phases for the model: phase 1 (May 16, 2009, through August 2, 2009) and phase 2 (August 3, 2009 through December 31, 2009). In addition, the consultation rate for ILI cases had changed over the course of the pandemic, because of changes in strategies used to control the disease in Beijing before and after National Day (October 1). To adjust for the introduction of these strategies, we further divided phase 2 into 2 periods: period 2a (from August 3, 2009, through September 30, 2009) and period 2b (from October 1, 2009, through December 31, 2009).

During phase 1, the number of laboratory-confirmed cases was considered to reflect the true number of pandemic (H1N1) 2009 infections. However, during phase 2, we calculated the true number of infections by multiplying the baseline by the estimation coefficient, using the multiplier model. In this multiplier model, the baseline case number was equal to the sum of the product of the weekly ILI case number in level 2 and 3 hospitals and the corresponding weekly pandemic (H1N1) 2009 positive rate among case-patients with ILIs. The estimation coefficient was found by multiplying the reciprocal of the parameters in the model. The following parameters were required in our estimation: the proportion of symptomatic infection among patients with cases of pandemic (H1N1) 2009, the proportion of ILI among patients with symptomatic cases of pandemic (H1N1) 2009, the consultation rate among ILI case-patients, the sampling success rate, and the sensitivity of the test (Figure; Table 1). These terms were obtained from a review of the literature (*8*–*12*) and from recommendations by health professionals. We assumed that the consultation rate of ILIs in each of the 2 periods was consistent and that the syndromic profile of pandemic (H1N1) 2009 did not change greatly.

**Figure:**
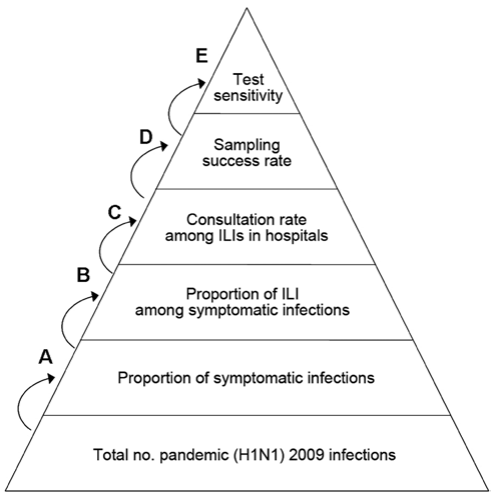
Model parameters for estimating the true number of persons infected with pandemic (H1N1) 2009 in Beijing. A, hospitals refer to level 2 and 3 hospitals in Beijing; B, sampling success rate was included in the model because not all actual positive specimens gave positive results because of the timing of collection or the quality of the specimen; C, test sensitivity was included in the model because not all actual positive specimens gave positive results due to the insensitivity of PCR reagent and unpredictable errors in experimental operations and instruments; D, proportion of true pandemic (H1N1) 2009 cases for which specimens were successfully collected; E, proportion of true positive specimens that were correctly identified by PCR reagent. ILI, influenza-like illness.

**Table 1:** Parameter values and sources of data included in the multiplier model for estimating the true number of persons infected with pandemic (H1N1) 2009, Beijing*†

Code	Parameter	Value, %	Source
A	Proportion of symptomatic infection among case-patients with pandemic (H1N1) 2009	70–75	Pandemic (H1N1) 2009, ECDC Risk Assessment.,2009; version 6, 6 Nov.
B	Proportion of ILI among symptomatic case-patients with pandemic (H1N1) 2009	26–42	Literature and unpublished clinical data
C1 (period 2a)	Consultation rate among ILI case-patients in secondary and tertiary hospitals	38	Telephone interview conducted by Beijing CDC
C2 (period 2b)	Consultation rate among ILI case-patients in secondary and tertiary hospitals	48	Telephone interview conducted by Beijing CDC
D	Sampling success rate	80–90	Previous surveillance data
E	Sensitivity of test	95–100	Professional recommendations

*ECDC, European Centre for Disease Prevention and Control; ILI, influenza-like illness; Beijing CDC, Beijing Center for Disease Prevention and Control. †The multiplier model was only used for phase 2 in this study, and phase 2 was divided into 2 periods, period 2a and period 2b. During phase 2, the true number of infections was calculated by multiplying the baseline by the estimation coefficient, using the multiplier model. The baseline case number was equal to the sum of product of weekly ILIs number in level 2/3 hospitals and the corresponding weekly pandemic (H1N1) 2009 positive rate among case-patients with ILIs. The estimation coefficient was obtained by multiplying the reciprocal of the parameters mentioned in this table. The baseline case numbers in periods 2a and 2b were 6,520 and 171,899, respectively.

In phase 1, a total of 325 positive cases were reported (considered as the true infection number). In period 2a and period 2b of phase 2, the baseline case numbers were 6,520 and 171,899, respectively. During phase 2, a total of 1,800,074 pandemic (H1N1) 2009 infections were estimated. Thus, by the end of 2009, the cumulative number of persons infected with pandemic (H1N1) 2009 in Beijing was estimated to be 1,800,399 (90% range 1.46–2.30 million) (Table 2). However, only 10,844 laboratory-confirmed cases were reported during the same period. One laboratory-confirmed case equaled 166 (90% range 135–212) infections in reality. According to the population size of Beijing, the overall infection rate was 10.6%. The highest infection rate was recorded in those 5–14 years of age (31.8%), followed by those 0–4 years of age (30.8%) (Table 2). In comparison, the rate in persons >60 years was only 0.9%.

**Table 2:** Estimated numbers of persons infected with pandemic (H1N1) 2009 and infection rate, by age group, Beijing*

Age group, y	Proportion of total no. persons infected, %	Estimated no. cases, median (90% CI)	Estimated rate, %, median (90% CI)
0–4	13.4	241,253 (195,910–307,571)	30.8 (25.0–39.2)
5–14	35.1	632,300 (513,459–806,111)	31.8 (25.8–40.6)
15–24	29.4	528,597 (429,247–673,902)	22.2 (18.0–28.3)
25–59	20.9	375,383 (304,829–478,571)	4.1 (3.3–5.2)
>60	1.3	22,865 (18,568–29,150)	0.9 (0.7–1.1)
Total	100.0	1,800,399 (1,462,012–2,295,305)	10.6 (8.6–13.5)

*CI, confidence interval.

## Conclusion

Despite the small number of laboratory-confirmed cases (10,844), we estimated that the actual number of persons infected with pandemic (H1N1) 2009 was 1.8 million in Beijing by the end of 2009. Previous studies have claimed that the number of laboratory-confirmed cases of pandemic (H1N1) 2009 was substantially underestimated, reflecting only a very small fraction of the actual infections (*3*–*5*). This study also demonstrated that school age children were more likely to be infected with pandemic (H1N1) 2009. However, those >60 years of age were at low risk for infection.

From November 27 through December 7, 2009, a serologic survey to establish the prevalence of pandemic (H1N1) 2009 antibody was conducted in the general population of Beijing. The results showed that ≈14%–15% (*13*) of the general population had antibodies to pandemic (H1N1) 2009 virus. Based on the population size of 17 million in Beijing in 2009 (*13*) and the assumption that antibodies against pandemic (H1N1) 2009 virus are usually produced after 2 weeks of infection or vaccination (*14*), we estimated that 2.37 to 2.54 million persons were infected with pandemic (H1N1) 2009 virus as of November 13, 2009. According to data from the Beijing Center for Disease Prevention and Control, by November 13, 2009, 1.36 million persons had received the pandemic (H1N1) 2009 vaccine. After the vaccinated population were removed from the equation, the total number of pandemic (H1N1) 2009 cases was estimated to be ≈1.01 to1.18 million. At the same time, the number of infections was estimated at 0.87–1.28 million as of November 13, 2009, by the multiplier model (data not shown in the section of the study). The estimates of the infection matched with the actual number estimated from the serologic survey in principle.

In phase 1, the number of laboratory-confirmed cases was considered to reflect the true infection number. This assumption, however, may lead to an underestimation for 2 reasons. First, we ignored the parameters used in phase 2, and second, difficulties occurred in testing all of the samples taken from patients who sought consultation for ILIs. Nevertheless, because the pandemic did not spread in the community in phase 1, we believe that this underestimation would have been quite low.

Although, in theory, serologic surveys should provide an accurate record of the infection rate of pandemic (H1N1) 2009, they failed to provide a quicker and more representative result than the multiplier model. Given the similarities between the estimates obtained from the model and the estimates obtained from the serologic survey, we conclude that the multiplier model based on the Monte Carlo approach should be considered a useful and simple method for estimating the true number of infections during a pandemic.
